# Designing Telemedicine for Older Adults With Multimorbidity: Content Analysis Study

**DOI:** 10.2196/52031

**Published:** 2024-01-10

**Authors:** Nida Buawangpong, Kanokporn Pinyopornpanish, Suphawita Pliannuom, Nopakoon Nantsupawat, Nutchar Wiwatkunupakarn, Chaisiri Angkurawaranon, Wichuda Jiraporncharoen

**Affiliations:** 1 Department of Family Medicine Faculty of Medicine Chiang Mai University Chiang Mai Thailand; 2 Global Health and Chronic Conditions Research Group Chiang Mai University Chiang Mai Thailand

**Keywords:** telemedicine, telehealth, chronic disease, multimorbidity, older adults, mobile phone

## Abstract

**Background:**

Telemedicine is a potential option for caring for older adults with multimorbidity. There is a need to explore the perceptions about telemedicine among older adults with multimorbidity to tailor it to the needs of older adults with multiple chronic conditions.

**Objective:**

This study aims to explore the perceptions about telemedicine among older patients with multimorbidity.

**Methods:**

A qualitative study was conducted using semistructured interviews. The interview questions examined older adults’ perspectives about telemedicine, including their expectations regarding telemedicine services and the factors that affect its use. Thematic analysis was performed using NVivo (version 12; Lumivero). The study was reported using the Standards for Reporting Qualitative Research guidelines.

**Results:**

In total, 29 patients with multimorbidity—21 (72%) female patients and 8 (28%) male patients with a mean age of 69 (SD 10.39) years—were included. Overall, 4 themes and 7 subthemes emerged: theme 1—perceived benefit of telemedicine among older adults with multimorbidities, theme 2—appropriate use of telemedicine for multimorbid care, theme 3—telemedicine system catering to the needs of older patients, and theme 4—respect patients’ decision to decline to use telemedicine.

**Conclusions:**

Telemedicine for older adults with multimorbidity should focus on those with stable conditions. This can help increase access to care for those requiring continuous condition monitoring. A structured telemedicine program and patient-centered services can help increase patient acceptance of telemedicine. However, health care providers must accept the limitations of older patients that may prevent them from receiving telemedicine services.

## Introduction

### Background

The COVID-19 pandemic has led to the emergence of telemedicine as a viable alternative to traditional, in-person care. Telemedicine has the potential to provide convenient medical care for patients with disabilities, transportation limitations, or busy schedules, enabling them to receive care from home [1-3]. It has become an increasingly valuable tool for delivering care to patients with multimorbidity, who require regular monitoring or adjustments to their treatment plans [4,5]. An emerging care model illustrates the integration of a patient-centered approach for individuals and chronic care model with multimorbidity. The model offers comprehensive care across various patient aspects and uses a multidisciplinary approach to address the complexity of managing multimorbidity [6]. Telemedicine can provide remote consultations and monitoring, provide patient education, and facilitate continuity of care [7,8].

In many countries, telemedicine has been promoted in the post–COVID-19 era owing to comparable health outcomes and favorable cost-effectiveness compared with in-person visits [9]. In Thailand, telehealth projects have been launched by the National Broadcasting and Telecommunication Commission of Thailand and the Thai Ministry of Public Health to improve health care services’ accessibility and quality [10]. As Thailand has become an aged society, with approximately 12 million people aged ≥60 years [11], telemedicine can help improve the quality of life of older adults by promoting healthy behavior, enhancing social functioning, and reducing depressive symptoms [12,13]. Telemedicine can also improve health for older patients, who often have multimorbidity, by helping to provide continuous medical care [14].

However, the lessons learned from using telemedicine during the COVID-19 pandemic have documented challenges for both older patients and health care providers (HCPs), as it was an unfamiliar mode of treatment compared with in-person service [15]. In addition, the literature suggests that older patients may prefer in-person visits owing to the frustration caused by technological challenges when using telemedicine [16]. However, their perceptions and preferences regarding telemedicine remain poorly understood. Therefore, the needs of older patients for telemedicine should be explored [17].

Moreover, there are gaps in understanding telemedicine for those with multimorbidity [6,18]. Many studies showed the effectiveness of telemedicine, but most focused on the use of telemedicine for a single disease [19-21]. Managing patients with multimorbidity is an increasing challenge in primary care practice [22]. In multimorbidity, there are many interactions such as disease-disease, treatment-treatment, and disease-treatment, which increase the complexity of management [23,24]. The rising question is what is the appropriate use of telemedicine in caring for older patients with multimorbidity [25].

### Objective

Our study aimed to explore the perceptions about telemedicine among older patients with multimorbidity. By understanding the perceptions about expectations, preferences, and barriers regarding telemedicine, the results can be used to develop telemedicine strategies to support the management of multimorbidity in the older population.

## Methods

### Study Design

A qualitative study of older adults with multimorbidity attending a primary care outpatient clinic at a university hospital in Thailand was conducted in 2021. The study used the Standards for Reporting Qualitative Research guidelines, which is a list of 21 items considered essential for complete, transparent reporting of qualitative research [26].

### Setting and Participants

The Family Medicine Clinic at the Faculty of Medicine, Chiang Mai University, is a primary care clinic that provides general medical care for chronic diseases. Approximately 80% of all patients are older adults (aged ≥60 y) with multimorbidity. Multimorbidity was defined as the patient’s illness that includes the presence of multiple diseases or conditions, often with a cutoff of ≥2 conditions [27]. The most common conditions are hypertension, type 2 diabetes mellitus, and dyslipidemia. Approximately 90% of the patients had multimorbidity. Among those attending the clinic, the rate of controlled hypertension, type 2 diabetes mellitus, and dyslipidemia between August and October 2021 were 93.7%, 70.3%, and 79.8%, respectively.

Convenience sampling was used. Patients aged ≥60 years and diagnosed with at least 2 chronic conditions who were accessible and available were invited to participate in the study. The patients needed to have stable conditions, defined as being asymptomatic and not having any urgency or emergency conditions, according to the national clinical guidelines for managing hypertension [28], type 2 diabetes mellitus [29], and dyslipidemia [30].

### Telemedicine Service

Telemedicine is the use of electronic information and communication technologies to provide and support health care when distance separates the participants [31]. In 2020, the Family Medicine Clinic in Thailand started a telemedicine service in response to the COVID-19 pandemic. After the COVID-19 pandemic, our facility continues to use telemedicine for delivering care to patients with chronic conditions. By considering patient safety, patients with moderate to well-controlled chronic conditions were approached to participate in the telemedicine service for continuous care [32]. In addition, it is essential to discuss with the patients the purpose of telemedicine and to address any limitations associated with its use [33]. In addition, the objective of telemedicine as ongoing care and limitations of telemedicine need to be discussed with the patients.

The clinic had adopted a published multimorbidity assessment checklist developed to help care for patients with multimorbidity [6]. The 20-item assessment checklist (Simple Multimorbidity Assessment Checklist for Primary Care) incorporates patient-centered concepts into managing multimorbidity in primary care settings, including assessment of the patient, review of all diseases and conditions, review of all treatments, review of clinical practice guidelines, assessment of interactions, understanding patient context and concerns, finding common ground, setting individual care plan, and continuity of care and follow-up visits. The checklist was also extended for use in the telemedicine service for assessing patients who were suitable for telemedicine and health caregiving.

The telemedicine service was provided via video or audio call, depending on the patients’ available devices and abilities. Physicians could collect patients’ medical histories and evaluate emergency or urgent conditions during the consultation. If any patient had conditions that required further evaluation, they were advised to come to the hospital. If patients did not require any further in-person assessment, medications were prescribed and delivered to the patient’s home via post.

### Ethical Considerations

This study was reviewed and approved by the institutional ethics committee of the Faculty of Medicine, Chiang Mai University, Chiang Mai, Thailand (approval number 227/2021). All participants were informed about the research study and provided consent.

### Data Collection and Analysis

Semistructured interviews were conducted between September and November 2021. The interview questions (Multimedia Appendix 1) focused on older patients’ perceptions about telemedicine, including their preferences regarding expectations from telemedicine services, and factors that affect its use, using the Unified Theory of Acceptance and Use of Technology framework [34]. This framework illustrates a comprehensive understanding of all the factors that affect people’s intentions to use the new technology. A research assistant, not involved in providing medical care, was trained in the interview method and interview questions by WJ and NB. Each interview lasted approximately 20 minutes and was conducted on-site. Interviewed information included baseline characteristics (age, sex, educational level, employment status, and their decision regarding telemedicine services) with permission for audio recording. Then, the interviews were transcribed verbatim. Data collection and analysis were performed iteratively by researchers. Recruitment ended when data saturation of the core analytic content had been achieved. Previous literature suggested that the sample size of 9 to 17 interviewees could help to reach saturation [35]. We further determined the sample size based on a previous study investigating the crucial factors for outpatient service selection among older adults. At least 16 patients were required to achieve data saturation [36]. Therefore, we considered collecting data from at least 16 patients until we achieved data saturation in the results [37].

Each transcript was evaluated multiple times to aid familiarization and understanding of the data. Descriptive analysis was used to describe the patient’s characteristics. For qualitative analysis, 2 independent researchers (NB and WJ) conducted inductive thematic analysis [38]. The preliminary results were then interpreted and discussed with KP, SP, NN, and CA. Codes were then developed based on patterns in the data. The identified codes were compared and discussed for similarities and differences until consensus was reached regarding the emergent themes and subthemes. Data analysis was performed using NVivo (version 12; Lumivero).

## Results

### Overview

In total, 29 older patients with multimorbidity participated. Of the 29 participants, 21 (72%) were women and 8 (28%) were men. The mean age was 71 (SD 7.17) years. The 2 most prevalent underlying diseases were dyslipidemia (27/29, 93%) and hypertension (25/29, 86%). Most patients had completed primary school (11/29, 38%) or had a bachelor’s degree (10/29, 35%). Of the 29 patients, 23 (79%) were retired and 6 (21%) were self-employed. Of the 29 patients, 18 (62%) patients were interested in using telemedicine, whereas 11 (38%) patients were not interested in telemedicine and rejected telemedicine when they were offered. Patients’ characteristics are summarized in Table 1.

From the semistructured interviews, 4 themes and 7 subthemes emerged. The themes and subthemes are summarized in Textbox 1.

**Table 1:** Patients’ characteristics (N=29).

Characteristics		Values
Age (y), mean (SD)		71 (7.17)
**Sex, n (%)**		
	Female	21 (72)
	Male	8 (28)
**Chronic conditions, n (%)**		
	Dyslipidemia	27 (93)
	Hypertension	25 (86)
	Type 2 diabetes mellitus	9 (31)
	Others	6 (21)
**Number of chronic conditions, n (%)**		
	2	19 (66)
	3	7 (24)
	4	1 (3)
	5	2 (7)
**Educational level, n (%)**		
	No education	1 (3)
	Primary school	11 (38)
	Secondary school	5 (17)
	Vocational certificate	1 (3)
	Bachelor’s degree	10 (34)
	Master’s degree	1 (3)
**Working status, n (%)**		
	Retired	23 (79)
	Self-employed	6 (21)
**Decision regarding telemedicine, n (%)**		
	Accept	18 (62)
	Decline	11 (38)

**Summary of themes and subthemes.**

**Theme 1: perceived benefit of telemedicine among older adults with multimorbidities**

- Convenient to access without the need for travel
- Minimize the risk of COVID-19 transmission

**Theme 2: appropriate use of telemedicine for multimorbidity**

- Telemedicine for monitoring stable conditions
- Enhancing the self-management of chronic conditions

**Theme 3: telemedicine system catering to the needs of older patients**

- Telemedicine services should be as similar as possible to in-person care
- Telemedicine services should adopt a clear protocol that includes in-person visits
- Supporting the development of technological skills and providing resources

**Theme 4: respecting patients’ decision to decline to use telemedicine for various reasons**

### Theme 1: Perceived Benefit of Telemedicine Among Older Patients With Multimorbidity

#### Overview

The benefits of telemedicine were collected from older patients. They reported the reasons why telemedicine should be used and its benefits. These include eliminating the requirement for travel and reducing the risk of contracting COVID-19.

#### Subtheme 1: Convenient to Access Without the Need for Travel

The most mentioned benefit of telemedicine was eliminating the need for travel. Participants mentioned that telemedicine is convenient for accessing and receiving continuity of care. It can also save time and money, such as time spent in driving and finding parking. A patient mentioned that telemedicine reduces stress from long wait times at hospitals. It also eliminates the risk of driving accidents, especially in older patients with sensory problems owing to physiologic changes. In addition, there is no burden on family members or caregivers to come and drop them at the hospital:

...If we compare the advantages and disadvantages, there are more advantages, as it saves both time and cost. We don’t have to drive, look for parking spots, or wait in line [to meet the doctor and receive medication]. The advantages are greater.

Participant 13; female; aged 64 years; teacher; uncontrolled hypertension and obesity

I like it because I don’t have to go to the hospital. It’s convenient. If I had to give [telemedicine] a score, it would be a ten because it’s convenient for me. I don’t have to drive there because I’m not good at driving right now. I have to ask my husband to take me there.

Participant 5; female; aged 72 years; housemate; well-controlled hypertension, type 2 diabetes mellitus, and dyslipidemia

Because we don’t have to go to the hospital anymore. They send the medication to our house. It’s difficult to go to the hospital now. We have to ask our children to take us, but everyone is working. I want to receive the medication at home because I take this medication regularly.

Participant 26; female; aged 72 years; retired; well-controlled hypertension, type 2 diabetes mellitus, and dyslipidemia

Sometimes, if I go to the hospital to see a doctor, I have to wait a long time, and it can be stressful. With video calls, I can talk for a long time.

Participant 23; male; aged 76 years; retired; uncontrolled type 2 diabetes mellitus, well-controlled hypertension, and dyslipidemia

#### Subtheme 2: Minimize the Risk of COVID-19 Transmission

Patients perceive telemedicine as a helpful way to reduce the risk of SARS-CoV-2 infection by avoiding contact with individuals with infection at the hospital. By not having to physically go to the hospital, there is no need to wait in crowded areas for a physician or medication after treatment, resulting in decreased rate of contact:

I don’t have to go to places with many COVID-19 cases. My daughter also likes it because I don’t have to take risks. Using telemedicine is very good for me.

Participant 2; female; aged 72 years; self-employed; well-controlled hypertension, type 2 diabetes mellitus, and dyslipidemia

### Theme 2: Appropriate Use of Telemedicine for Multimorbidity

The participants felt that telemedicine should be used to care for patients with stable conditions in evaluating, monitoring, and providing health promotion. Participants also acknowledged the limitations of telemedicine in providing medical care, such as the inability to perform a complete physical examination or blood tests.

#### Subtheme 1: Telemedicine for Monitoring Stable Conditions

Patients feel confident in receiving telemedicine services when they have stable conditions because they have no abnormal symptoms, and the on-site care provided is only in the form of conversation to monitor their condition. Telemedicine services for those with stable conditions can resemble on-site care. Telemedicine services should include monitoring of clinical symptoms, vital signs, body weight, and behavioral factors. Consultation time is also required, so that patients can consult with their physician and inquire about their condition and receive follow-up care:

It’s just like when we see a doctor at the hospital. If we meet the doctor, we ask questions like this. We can also ask online like this and see each other’s faces; finding a doctor this way is good and convenient. The doctor called, and we talked. If we have any questions, we ask, and the doctor answers. It’s just like going to see a doctor.

Participant 18; female; aged 69 years; retired; well-controlled type 2 diabetes mellitus and osteoporosis

However, some patients still believe that if they experience new or more severe symptoms, they prefer to receive treatment in an in-person setting for more detailed examinations or blood tests:

It’s [telemedicine] comprehensive, but only if I do not have severe symptoms...However, if the patient has more severe symptoms..., it’s uncertain how effective the treatment [received through telemedicine] will be.

Participant 19; female; aged 66 years; retired; well-controlled hypertension and type 2 diabetes mellitus

It’s [telemedicine] good. Luckily, there have been no issues during this period. But if any problems arise, I still have to go see a doctor.

Participant 15; female; aged 73 years; retired; uncontrolled type 2 diabetes mellitus and well-controlled hypertension

#### Subtheme 2: Enhancing the Self-Management of Chronic Conditions

The patients perceive that they can take better care of themselves when telemedicine provides health care information specific to their health problems. Some patients suggested incorporating self-monitoring and health promotion features into the telemedicine platform. They believe in sharing self-management information with physicians to improve disease management, such as home monitoring of blood pressure and blood sugar levels. The platform could also provide specific knowledge for lifestyle modification, such as exercise videos or electronic brochures about food exchange lists. This enhances the potential for self-management:

I normally check and record my blood sugar and blood pressure at home. Sometimes, I forget to bring the records to the hospital. However, in telemedicine, when the doctor calls, I can inform them of my records.

Participant 2; female; aged 72 years; self-employed; well-controlled hypertension, type 2 diabetes mellitus, and dyslipidemia

I want to know more about blood pressure. We may think it’s not a big deal, but actually, it’s a silent danger. The doctor said it’s a scary disease, and I want information on how to take care of this disease. It would be great if there were some tips on this matter.

Participant 14; female; aged 66 years; government employee; uncontrolled hypertension and well-controlled type 2 diabetes mellitus

If there is a LINE group [chat group], I would like to receive informative messages about health. Even seniors need to read news and information related to health, such as knowledge about [COVID] vaccines.

Participant 21; female; aged 75 years; retired; well-controlled hypertension and type 2 diabetes mellitus

### Theme 3: Telemedicine Services Catering to the Needs of Older Patients

For an effective telemedicine service for older patients, it is crucial to address patient concerns and establish a clear management protocol based on their health status. Providing supporting resources, having good communication skills, and being aware of potential barriers arising from unfamiliarity with technology are also essential in meeting their needs.

#### Subtheme 1: Telemedicine Services Should Be as Similar as Possible to In-Person Care

When using telemedicine, patient concerns must still be evaluated, similar to in-person service. Some concerns may persist even after receiving telemedicine services. Presenting conditions can influence their physical or mental well-being, making it crucial for HCPs to thoroughly understand patients’ illnesses to ensure appropriate management. Some patients feel that video call feels more similar to an actual on-site visit than audio calls because they can see the facial expressions and gestures of the HCPs:

Video calls would be better because the doctor can see the patient’s face and how they feel at that moment, whether they are feeling stressed or not. With video calls, I feel closer [to the doctor] and more comfortable.... I usually don’t share things within my family unless it’s with the doctor. But with video calls, I feel more comfortable because I can see the doctor’s face, knowing that they care about me. I just want the doctor to call me and ask what I want to share or talk about.

Participant 1; female; aged 62 years; retired; well-controlled hypertension and type 2 diabetes mellitus

With video calls, we feel close to each other. It feels like we’re still talking to each other. It’s good because we can talk to the doctor about anything comfortably. I think it’s a good thing because patients can express themselves fully to the doctor.

Participant 17; male; aged 76 years; retired; well-controlled hypertension and type 2 diabetes mellitus

#### Subtheme 2: Telemedicine Services Should Adopt a Clear Protocol That Includes In-Person Visits

Patients express concerns that telemedicine might replace traditional, in-person care, leading to a lack of access to physical examinations, blood tests, and additional symptom management. They desire telemedicine to complement a comprehensive multimorbidity management program while still having the option to see physicians in person at the hospital:

Sometimes I want to meet with the doctor in-person to talk directly or ask questions. The doctor can know my symptoms if I communicate directly. If I say that it hurts here or it is swollen here, the doctor can touch it and examine it for evaluation. This is the basic step of diagnosing symptoms. I mean, I want to meet the doctor sometimes, but not frequently. It’s not like I don’t see the doctor for a year. I just want to see the doctor once or twice to feel reassured.

Participant 16; female; aged 74 years; retired; well-controlled hypertension, type 2 diabetes mellitus, and dyslipidemia

The support system of telemedicine services was crucial for patients’ decision-making regarding whether to accept or decline the service. Most patients are willing to accept telemedicine owing to clear operational systems, including appointment scheduling, notifications, web-based payment, and medication delivery. However, some patients still have doubts about payment systems and medication delivery. In addition, patients receiving telephone-based care may have uncertainties about the authenticity of the HCP:

After a case manager added me on LINE [application], they gave me an appointment for a video call. A day before the appointment, a nurse called me and said the doctor would have a video call tomorrow. When the appointment arrived, they would call me and ask if it was convenient for the doctor to have a video call now. I answered that it was. Then, the doctor called me. It was a very good process.

Participant 15; female; aged 73 years; retired; uncontrolled type 2 diabetes mellitus and well-controlled hypertension

It’s possible to send the appointment time through LINE in advance; for example, if the doctor would come in the afternoon, someone would call in the morning to inform. The doctor could then ask about the symptoms very well. It was done quickly, in just a moment.

Participant 21; female; aged 75 years; retired; well-controlled hypertension and type 2 diabetes mellitus

I asked about the cost of the medicine that sent text information by phone because I couldn’t contact the finance department. It’s very difficult.

Participant 18; female; aged 69 years; retired; well-controlled type 2 diabetes mellitus and osteoporosis

I don’t know if they are doctors or not. To be honest, I don’t know who is calling me. But if it’s a video call, I can be more confident.

Participant 13; female; aged 64 years; teacher; uncontrolled hypertension and obesity

#### Subtheme 3: Supporting the Development of Technological Skills and Providing Resources

Most participants had limited technological skills and relied on their children to assist in using electronic telemedicine devices. Only a small minority were proficient in using such devices, with some preferring mobile phone calls over video calls owing to incompatible smartphones or unfamiliarity with more complex devices such as tablets or PCs. However, some older individuals expressed willingness to learn with proper support:

If necessary, I need to adapt. I have to learn to use additional equipment because I don’t usually have a smartphone, so it might be difficult to learn. I can’t even turn it on.

Participant 9; female; aged 78 years; retired; well-controlled hypertension, type 2 diabetes mellitus, and dyslipidemia

If it’s time for telemedicine, I have to try to adapt and learn gradually.

Participant 10; female; aged 71 years; retired; well-controlled hypertension, type 2 diabetes mellitus, and dyslipidemia

### Theme 4: Respecting Patients’ Decision to Decline to Use Telemedicine for Various Reasons

Older patients often reject telemedicine owing to various obstacles. They face challenges related to age-related physiological changes, including forgetfulness and cognitive difficulties such as finding phones or using video calls despite instructions. In addition, some patients do not regularly use electronic devices, whereas others feel burdensome relying on their children for telemedicine assistance. A few patients are unable to receive telemedicine service owing to their routine work commitments. Therefore, HCPs need to understand these obstacles and respect their decisions for declining telemedicine:

I don’t want to use telemedicine. It’s not difficult for me to see a doctor in person. If you teach me something, I will forget in three months. For example, when I wanted to take a video, my grandchild had to teach me ten times, but when I got home, I couldn’t remember. My memory has not been good for a few years.

Participant 12; male; aged 68 years; self-employed; well-controlled hypertension and type 2 diabetes mellitus

Sometimes I am not with my phone. Like when I went to a restaurant, I forgot my phone there and didn’t realize it for three days. I think it’s a problem related to age, but if I were newly retired at 60-65 years old, I would be fine. But now that I’m nearly 80, I have problems, especially with memory and internet use.

Participant 23; male; aged 76 years; retired; well-controlled hypertension and type 2 diabetes mellitus

If my child can help, that would be great. I need my child to be here because I don’t know much. I’m forgetful, but I don’t know if my child is available to help or not. I can’t do it if I’m alone because I have to care for two other older people who are 90 years old. It’s not easy for me because I have to take care of others as well.

Participant 4; female; aged 63 years; retired; uncontrolled type 2 diabetes mellitus, well-controlled hypertension, and dyslipidemia

I can participate, but I’m not familiar with it. I’m old and have never used LINE [chat application] before. Trying to learn it now may be difficult because my memory is not very good, and I tend to forget things easily. Although my grandchildren have computers and mobile phones, I don’t want to bother them because they have to work all the time. If the doctor needs to call me, I have to ask my children. I don’t know if they’re available to answer or not.

Participant 8; female; aged 71 years; retired; uncontrolled type 2 diabetes mellitus and well-controlled dyslipidemia

## Discussion

### Principal Findings

#### Summary

In this qualitative study, participants perceived telemedicine as beneficial because it eliminates the need for travel and minimizes the risk of COVID-19 transmission. Older adults view telemedicine as a safe and effective way to manage stable chronic conditions. It is recommended to include health promotion in telemedicine services to enhance self-management. Regarding catering to older patients’ needs, patients expressed that telemedicine should be presented as part of a continuous care program for multimorbidity, incorporating web-based monitoring with periodic in-person visits for physical examinations and laboratory screenings. Clear instructions, technological skills training, and access to resources such as equipment and caregivers are essential to make the program user-friendly for older patients. However, HCP should respect patients’ decision to decline telemedicine owing to various obstacles that older patients may face when using it.

Patients accepted telemedicine as an effective method to improve access to health care for older patients. Some older patients face difficulties when coming to hospitals, such as finding transportation and parking space, long waits for the physician, and long queues for receiving medication [16,36]. This is in accordance with the literature that positive perceptions about telemedicine include cost savings [39]. During the COVID-19 pandemic, receiving treatment through telemedicine services also helped to reduce the risk of infection transmission by reducing the risk of overcrowding of patients and the risk of exposure for those who may not need to come to the hospital [40]. Telemedicine would be a necessary solution for addressing problems regarding access to care in the event of new pandemics.

Several studies have shown that telemedicine can effectively improve the health care outcomes of older patients, particularly those with chronic conditions such as diabetes [19], heart disease [20], and asthma [21]. A study found that telemedicine consultations reduced hospitalization rates among older patients with chronic heart failure compared with standard care [41]. Another study found that telemedicine consultations for older patients with chronic obstructive pulmonary disease improved symptom control and quality of life and reduced hospitalization rates [42]. There is evidence supporting that telemedicine services have the potential to enhance self-management among patients and their families, including improving medication adherence among older patients with chronic conditions and improving disease control and patient satisfaction [43,44].

Older patients, who often have >1 chronic condition, constitute a key group who use the health systems [45]. Owing to the situation in Thailand, it will be a superaged society in the next few decades [46]. The older adults would be the main target for health care delivery. There are potential opportunities in digital health such as telemedicine, emphasizing the management of chronic diseases in Thailand [47]. Cost-effectiveness was also another reason in the long run for telemedicine compared with an in-person visit [4,48]. On the basis of patients’ perceptions obtained from our study and previous evidence supporting the health outcomes of telemedicine, we have the following 4 suggestions for enhancing telemedicine services tailored to the needs of older patients with multimorbidity.

#### Identify the Target Population as Individuals With Stable Chronic Conditions

Our study found that telemedicine is an accepted model for promoting continuous care for older patients with multimorbidity. In cases where patients have well-controlled chronic conditions and no abnormal symptoms, they can receive symptom monitoring and treatment through telemedicine [49]. Health care services for older patients with stable chronic diseases may not need to differ between telemedicine and in-person visits. The services should aim to monitor patients with stable conditions by regularly inquiring about their symptoms; offering self-care instructions at home (such as measuring blood pressure and blood sugar levels); and encouraging healthy behaviors such as medication adherence, maintaining a proper diet, and engaging in regular exercise. Telemedicine has the potential to replace nonurgent in-person medical visits for stable chronic diseases, as it can be used for symptom monitoring, detecting complications or disease progression, and prescribing medication delivery for stable chronic conditions [7,50].

#### Telemedicine Services Should Be Designed to Closely Resemble In-Person Visits With Scheduled Periodic In-Person Visits

Patients expressed concerns regarding the quality of care and maintaining the physician-patient relationship received through telemedicine. A previous study revealed that older patients perceived in-person visits as fostering a strong physician-patient relationship compared with telemedicine [16]. To address these concerns, telemedicine services should strive to deliver care that closely resembles an in-person visit in terms of the process and pattern of care. Telemedicine using video calls closely simulates an in-person visit over phone calls. It helps reduce medication errors, enhances diagnostic accuracy, and improves decision-making accuracy [51]. Telemedicine holds the potential to facilitate shared decision-making between patients and HCPs, thus promoting a patient-centered approach to care [52]. HCPs can also leverage telemedicine to provide education, promote behavior change, empower patients to take control of their health, boost their confidence, and ensure continuity of care [53].

Nevertheless, it is important to acknowledge the limitations of telemedicine. It is unable to perform comprehensive physical examinations and detailed laboratory tests, which means that it cannot fully replace in-person visits. Hence, it is crucial to integrate regular in-person visits with a physician at a hospital to adhere to standard medical practices. These in-person visits can be scheduled periodically on an annual basis, which can help instill confidence and satisfaction with the telemedicine services [54].

#### Integrated Support Systems for Telemedicine, Including Clear Protocols, Caregiver Assistance, and Electronic Health Literacy Training

When caring for older patients with multimorbidity through telemedicine, it is essential to establish a program that adheres to standard practices, incorporates clear protocols, and provides the necessary technical skills and resources. This will help ensure that telemedicine remains as a viable option beyond the COVID-19 pandemic, emphasizing ease of use and demonstrating its benefits. A well-defined service program enables patients to understand the process of care they will receive and empowers them to communicate their specific health needs [55]. In addition, telemedicine services for older adults may require assistance at various stages, including guidance for using tools such as smartphones, instruction for use, and involving caregivers in the process [56]. These supports could contribute to a smooth and more effective telemedicine experience for older patients and their caregivers.

#### Develop Alternative Services for Older Adults Who Cannot Use Telemedicine

Despite the potential benefits of telemedicine for the care of older adults, its use has some challenges and limitations. Some older patients may face various obstacles in using telemedicine, such as declining vision, hearing, and memory owing to aging; difficulty in learning new skills; unfamiliarity with technology; and feeling burdened to ask for support, which can lead to rejecting telemedicine services [56]. In addition, telemedicine is unsuitable for unstable patients requiring emergent management and detailed physical examinations that cannot be conducted remotely [57]. Some specific clinical contexts or onset of new symptom in multimorbidity, such as hemiparesis, require a comprehensive examination for critical diagnostic accuracy and severity evaluation. If possible, an in-person visit would be more appropriate [58]. Thus, in-person visits or other alternatives should be available [7].

### Strengths and Limitations

The study has several strengths and limitations. The strength of this study lies in its structured approach to gathering insights about various aspects of telemedicine service tailored to the need of older adults with multiple chronic conditions. The study results can provide valuable guidance about preparing and delivering telemedicine services for this population. However, there are still some limitations to be considered. First, participants were recruited from a single health care facility. The results may be affected by the nature of the health care system and the educational level and digital literacy level of the population. Further studies from different settings and regions are needed to tailor telemedicine services to the needs of older adults with multimorbidity. In addition, future studies could explore more experiences of HCPs providing telemedicine services to this population and identify strategies to address their challenges and concerns. Next, we did not include uncontrolled conditions in this study, and we did not include health care professionals. Further studies may include other telemedicine users. Another consideration point that could influence the results of this study is gender. Gender is associated with differences in digital health care behavior and plays a role in the adoption of health technologies. For example, women exhibit high tendency to access health care services, book physician’s appointments, and search for nutrition-related information. In contrast, men are more likely to explore options related to smoking cessation and use health apps for monitoring sleep patterns and blood pressure than women [59].

As there are various guidelines for telemedicine management, the platform used depends on the facility in each hospital setting. Having many telemedicine providers may disrupt the continuity of care owing to regulation and personal data protection [60]. Furthermore, there remains an inequity in access to care in telemedicine for vulnerable population, such as older people. A strategy to promote electronic health education and provide the necessary equipment to ensure telemedicine equity is needed [61]. Therefore, the use of telemedicine should be tailored depending on the setting and needs of the population and health system.

To ensure the sustainability of telemedicine for older patients with multimorbidity, HCPs should consider patients’ needs, expectations, and abilities when designing telemedicine systems. Importantly, the findings also suggest that HCPs should respect the decision of older patients who decline to use telemedicine owing to multiple obstacles and find alternative ways.

### Conclusions

The study highlights the importance of personalized and patient-centered care [62], where providers should understand older adults’ needs, preferences, and limitations to tailor telemedicine services for the population. The use of telemedicine for older patients with multimorbidity should focus on those with stable conditions. For this population, telemedicine can help increase access to medical services for patients who require continuous monitoring and care. A structured program incorporating periodic in-hospital visits can help increase patient acceptance of telemedicine. However, HCPs must also understand the limitations of older patients owing to various factors that may prevent them from receiving telemedicine services.

## Appendix

Multimedia Appendix 1 Interview questions.

## Abbreviations

HCP: health care provider
