# Supplementary material for: Designing Telemedicine for Older Adults With Multimorbidity: Content Analysis Study
Source: JMIR Aging. 2024 Jan 10;7:e52031. doi: 10.2196/52031 (PMC10809167; doi:10.2196/52031)
Supplement: Multimedia Appendix 1 [file aging_v7i1e52031_app1.docx]

**Appendix: The interview questions**

**Section 1: For all participants**

**Factors influencing behavior and intention to use technology-mediated services include:**

**Performance Expectancy:** Do you believe that telemedicine services can effectively address your health concerns? Why or why not?

**Effort Expectancy:** Do you find telemedicine services difficult or easy to use? If applicable, rate the difficulty of using telemedicine services.

**Social Influence:** What influenced your decision to use telemedicine services?

**Technology Concerns:** What device (mobile, tablet, computer) do you use for telemedicine services? Do you have concerns about using the [device]? If yes, why?

**Resistance to Change:** If health care is only available through telemedicine, how willing are you to adapt? Do you think you can adapt to the changes?

**Section 2: For patients who are interested in using telemedicine**

**Access to Care/Service:** How do you access telemedicine care? Who invited you to participate? How convenient or inconvenient is technological access?

**Operations and Stakeholders:** How are you being cared for? Is the care comprehensive? How trustworthy and understandable is it? Are there areas where you feel neglected?

**Follow-up:** How are you being followed up by the care team?

**Satisfaction:** Are you satisfied with the telemedicine care system? Why or why not? (Rate if possible)

**Limitations:** What limitations do you perceive in telemedicine services? What additional support do you need, both in terms of resources and relevant skills?
